# The Influence of Skin Microcirculation Blood Perfusion at Zusanli Acupoint by Stimulating with Lift-Thrust Reinforcing and Reducing Acupuncture Manipulation Methods on Healthy Adults

**DOI:** 10.1155/2013/452697

**Published:** 2013-03-12

**Authors:** Xiaomei Li, Yanqi Li, Jingzi Chen, Dan Zhou, Yangyang Liu, Yinghong Li, Jianwei Liu, Yongming Guo, Yi Guo

**Affiliations:** ^1^Experimental Acupuncture Research Centre, Tianjin University of Traditional Chinese Medicine, Tianjin 300193, China; ^2^Department of Rehabilitation and Health Care, Hunan Traditional Chinese Medical College, Zhuzhou 412012, China; ^3^Department of Anatomy, Tianjin University of Traditional Chinese Medicine, Tianjin 300193, China

## Abstract

*Background*. In traditional Chinese medicine acupuncture manipulation is one of the key factors that affect the curative results of acupuncture and more and more researches focus on how the different acupuncture manipulation techniques influence microcirculation nowadays. In this paper we demonstrate the different influences of lift-thrust reinforcing and reducing on blood perfusion. *Method*. The acupuncture manipulations of lift-thrust reinforcing and reducing were, respectively, applied to the 15 healthy subjects at the Zusanli acupoint and the changes of blood perfusion were monitored by Pericam Perfusion Speckle Imager (PSI). *Conclusion*. Both of the manipulations of lift-thrust reinforcing and reducing increase blood perfusion at Zusanli acupoint while the increasing amount of blood perfusion in the reinforcing group is significantly higher than in the reducing group.

## 1. Introduction

In recent years, more and more patients in western countries turn to complementary and alternative medicine therapies [1]. Acupuncture especially is one of the most frequently requested of the complementary therapies [2]. According to a national cross-sectional survey, acupuncture provides a substantial contribution to the healthcare [3]. Acupuncture is increasingly used in managing chronic pain and other conditions, such as chronic knee pain, tension-type headache, low back pain, and so on [4–6]. Though it has clinical efficacy and cost-effectiveness, western medical experts have been skeptical of acupuncture's therapeutic value. Acupuncture rational basis underlying its use remains unclear [7]. In traditional Chinese medicine (TCM), acupuncture manipulation is one of the key factors that affect the curative results of acupuncture [8]. In Qing Dynasty, Li Shouxian said “It's not difficult to understand acupuncture point, but to master the manipulation; it's hard for us to master the manipulation more than to understand acupuncture point; if only you merely comprehend acupuncture point, you couldn't be a distinguished doctor.” He stressed the importance of acupuncture manipulation. In order to apply appropriate acupuncture therapeutic effectiveness, different acupuncture manipulations are required after needle insertion in clinical practice [9]. These manipulations are performed very widely ranging from reinforcing and reducing the needle to twirling the needle and varying the insertion angle, and so forth. With the continuous development and progress of science and technology recently, the research approach and methods of acupuncture manipulation continue to broaden and update. Many scholars adopt a multidisciplinary, multichannel method to carry out acupuncture reinforcing and reducing manipulations to obtain some meaningful results [10]. Applying different acupuncture manipulations, the degree of nervous excitement, the local oxygen tension, concentration of chemical substances, and the degree of temperature could be changed at point's area [10–13]. Li et al. reported that reinforcing and reducing methods can produce different effects on skin temperature [11]. Some scientists collected different acupuncture manipulation parameters and stabilization of mathematical model [14, 15]. In our lab in 2011, using the parameter tester ATP-II of acupuncture manipulation produced by Shanghai University of TCM we collect the experts acupuncture operation parameters (cycle, frequency, rising time of waveform, falling time of waveform, platform of rising wave, platform of falling wave, rising slope of waveform, falling slope of waveform, increased amplitude of waveform, and decreased amplitude of waveform) of reinforcing lifting and thrusting, reducing lifting and thrusting, establish databases, and build the corresponding mathematical model with the Fourier transform and kernel regression mathematical method. The microcirculation blood perfusion as an important indicator of energy metabolism has attracted the attention of researchers [16–21]. Skin microcirculation is a good indicator of presenting the effect of acupuncture. Different methods of acupuncture on acupoint can cause change in skin blood perfusion [22]. As for the study, we also take the effect of different acupuncture manipulations (e.g., reinforcing lifting and thrusting, reducing lifting and thrusting, even reinforcing-reducing method, and acupuncture group without manipulations) on the blood perfusion of offside Zusanli (ST36) acupoint as the basis of this experiment, in order to observe the influence of different acupuncture manipulations.

## 2. Methods

### 2.1. Ethics Statement

This study was reviewed and approved by the Institutional Review Board at the Institute of Acupuncture and Moxibustion, Tianjin University of TCM. Each participant read and signed an informed consent form.

### 2.2. Subjects

The study was conducted at Experimental Acupuncture Research Centre of Tianjin University of TCM between May 2012 and August 2012. Fifteen healthy volunteer students (7 men and 8 women) aged 25.4 ± 0.99 years (mean ± SD; range, 24–27 years) were recruited in this study, who had no history of diseases and had not taken any medicine 1 month before the experiment. Each subject had an adequate understanding of the procedure and purpose of this study. Throughout the experiment, the subjects were neither told nor able to see or hear any indication of which needle manipulation type was being performed. There are five groups as follows: control group, acupuncture group without manipulations (no manipulation), reinforcing lifting and thrusting group (reinforcing), reducing lifting and thrusting group (reducing), and even reinforcing-reducing method group (even). Each group has 15 persons with self-control method, and everyone accepted the above 5 kinds of manipulation methods.

### 2.3. Procedures

#### 2.3.1. Protocol for Experimental Conditions

**Figure 1:**
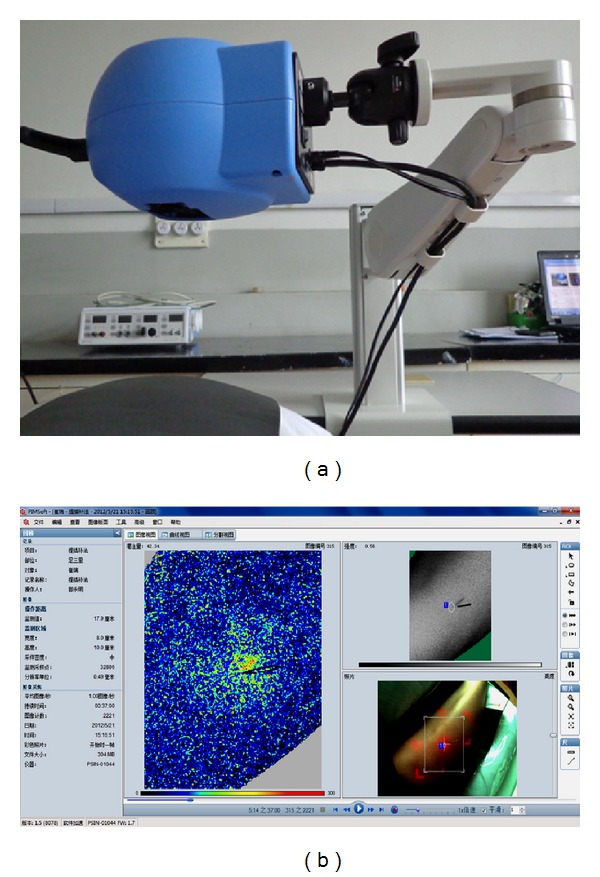
(a) Pericam Perfusion Speckle Imager (PSI). (b) The blood perfusion image of the monitor area. The brighter area is the definition of the Region of Interest (0.5 cm around the Zusanli acupoint).

The room temperature during the experiment was controlled at about 26 ± 1°C, and relative humidity was maintained 50%–60%, and there was no direct sunlight indoor. The PSI parameter was set as follows: image acquisition rate, 50 HZ; normal resolution, 0.5 mm; 1 frame per second; the working distance is 18 ± 1 cm; the monitor area is 10 (highness) × 8 (width) cm^2^; the definition of the Region of Interest (ROI) is in 0.5 cm around the Zusanli acupoint. PSI System is using the PIMSoft software for recording, saving, and analysis. PSI can dynamically and instantly monitor the change of blood flow perfusion in the body and display the image and blood flow curve at the same time. It also has the functions of video broadcast and output. Perfusion Unit (PU) is the unit used for blood perfusion. The higher the PU is, the greater the blood perfusion is. Right side of ST36 (Zusanli acupoint) is based on the national standard name and location (GB/T12346-2006). The sterile disposable needle used in acupuncture is 40 mm in length and 0.3 mm in diameter (Han Yi, Tian Jin).  Figure 1 shows Pericam Perfusion Speckle Imager (PSI) and the blood perfusion image of the monitor area.

#### 2.3.2. Operation Rules

Reinforcing group: insert the needle at offside ST36 and do the thrust heavily and lift lightly for 2 minutes after Deqi. Reducing group: insert the needle at offside ST36 and do the thrust lightly and lift heavily for 2 minutes after Deqi. Even group: insert the needle at offside ST36 and do even lifting, thrusting, and rotating for 2 minutes after Deqi. No manipulation group: insert the needle at offside ST36 without any manipulation. Control group: all subjects maintained still, without any intervention. Deqi is believed to be essential for efficacy of acupuncture according to TCM [23, 24].

**Figure 2:**
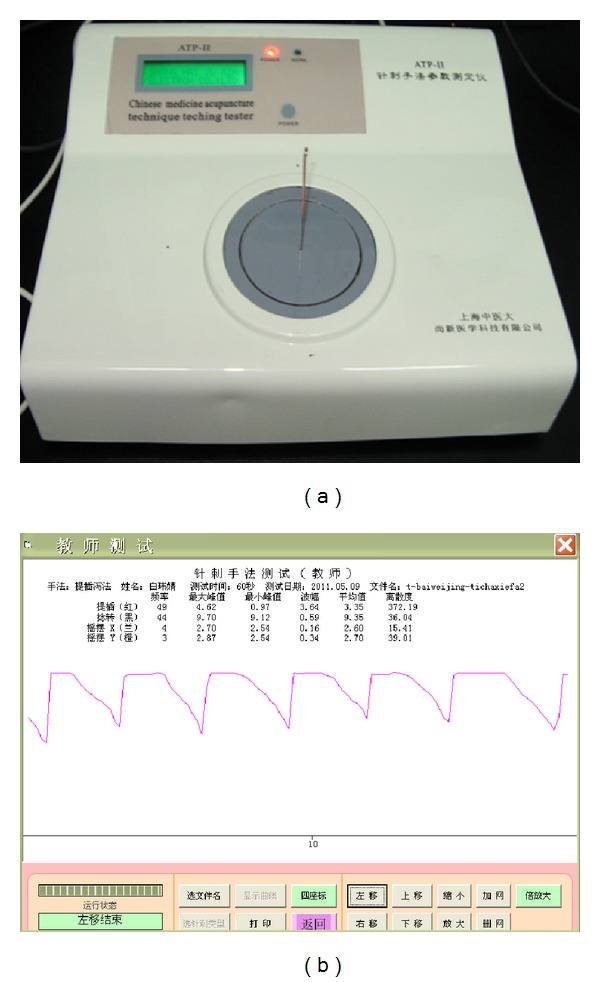
(a) ATP-II acupuncture manipulation parameter tester. (b) Output curve of acupuncture manipulation.

**Figure 3:**
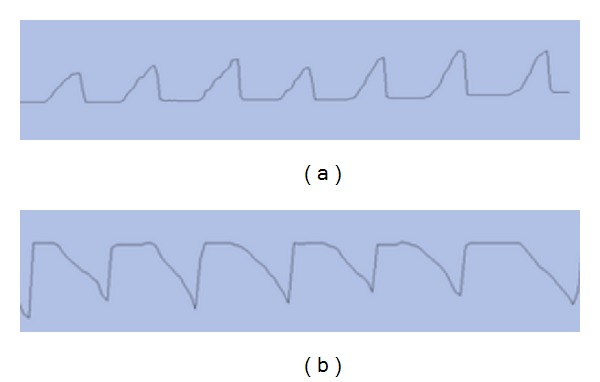
(a) Reinforcing manipulation by lifting and thrusting. (b) Reducing manipulation by lifting and thrusting.

To make sure of the stability of the acupuncture manipulation, before the experiment begin, the operator has had an operation skill training on the ATP-II acupuncture manipulation parameter tester (which was manufactured by Shanghai University of Traditional Chinese medicine ShangXin medical technology company). We compared the curve charts the operator made with the standard curve charts. The operator did not start the experiment until he reached the standard level. All the acupuncture manipulations during the whole experiment were made by the same person. This ensured consistent experimental conditions and eliminated many potential sources of investigator bias. The preliminary experiment showed that the blood perfusion would return normal 24 hours after needling on right side of ST36. To make sure that the effect of acupuncture disappeared, the operator did each manipulation at least two-day interval, at most three-day interval, and the experiment was done at the same time in different days.  Figure 2 shows the acupuncture technique parameter tester and the output curve.  Figure 3 shows the curves of the reinforcing manipulation and the reducing manipulation made by the operator. 

#### 2.3.3. Experiment Flow

**Figure 4:**
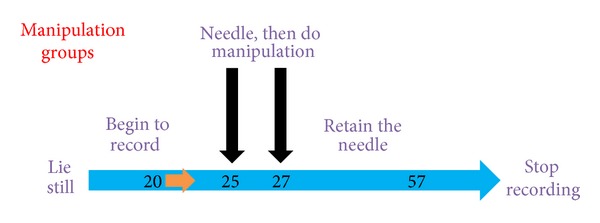
Procedure of acupuncture manipulation groups.

Every subject lies still for about 20 minutes for acclimatization. Then, skin at Zusanli acupoint was disinfected with alcohol. The control group blood perfusion was monitored about 35 minutes; data of the no manipulation group was recorded for 5 minutes before needling, and 30 minutes after needling; data of acupuncture groups with manipulations (including reinforcing manipulation by lifting and thrusting group, reducing manipulation by lifting and thrusting group, and even reinforcing-reducing manipulation group) were recorded for 5 minutes before needling, and 30 minutes after doing manipulations. The manipulation groups flow diagram is illustrated in Figure 4.

#### 2.3.4. Statistical Analysis

Statistical analyses were all performed in R statistical software. Repeated measurement ANOVA was used to assess the differences in mean blood perfusion between different groups and within single group. The trend over time was shown by the mean and standard deviation. At the same point-in-time, *t* test is used to find the significant difference.

## 3. Results

### 3.1. Study Participants

15 volunteers were enrolled in the study. All the participants completed the testing protocol.

### 3.2. Images

**Figure 5:**
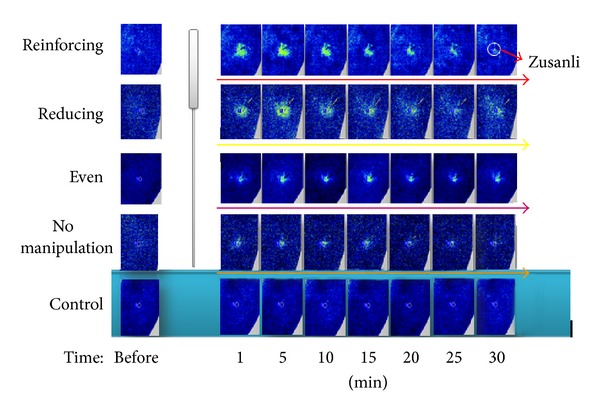
The blood perfusion images of different groups. As the blood perfusion is higher, the brighter areas indicate higher blood perfusion.

Figure 5 is composed of the blood perfusion images. It shows the changes of blood perfusion unit in different groups; the brighter areas indicate higher blood perfusion.

### 3.3. The Overall Trend of the Blood Perfusion Ratio in Different Groups

**Figure 6:**
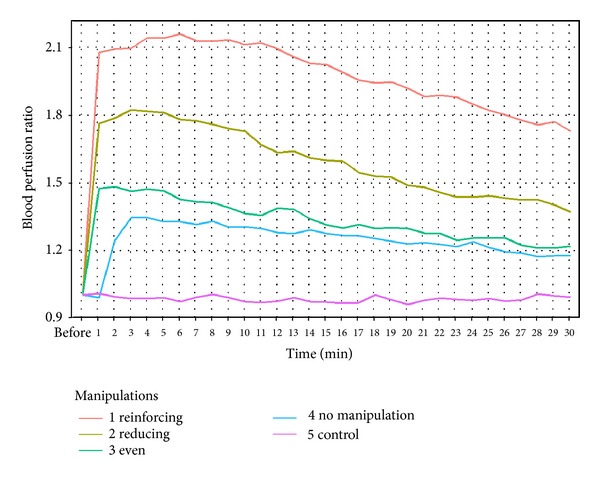
The overall trend of the blood perfusion ratio in different groups.

One data was recorded at every second. Every subject from each group was recorded for 35 minutes to get 2100 data as *x*_*ijk*_ (*i* = 1,…, 15 as subjects, *j* = 1,…, 5 as groups, *k* = 1,…, 2100). To every subject from each group: Step 1, average data for the first 5 minutes (before needle in acupuncture groups including manipulation groups and no manipulation group) as *X*_*ij*0_, *X*_*ij*0_ = ∑_*k*=1_^300^ *x*_*ijk*_. Step 2, average data in every minute after the first 5 minutes as *X*_*ijl*_, *X*_*ijl*_ = ∑_*k*=60×(*l*−1)+301_^300+60×*l*^ *x*_*ijk*_, *l* = 1,…, 30. Considering the circumstances for every subject are not exactly the same, we focus on the ratio between *X*_*ijl*_ and *X*_*ij*0_ and assume *Y*_*ijl*_ = *X*_*ijl*_/*X*_*ij*0_, *Y*_*jl*_ = ∑_*i*=1_^15^ *Y*_*ijl*_, and *l* = 1,…, 30. For each *j*, the time series (*Y*_*j*1_,…, *Y*_*j*30_) stand for the trend of one group.  Figure 6 shows the overall trend of the blood perfusion ratio in different groups.

From this we can see that all groups have similar changes, except the control group. First the PU rises obviously when the acupuncture stops; then it falls down in different periods of the trail. Furthermore, the trend of the PU falls down at different times in the four groups. The PU of the reinforcing manipulation by lifting and thrusting group is the most obvious because it could reach 2~2.5 times higher than the basic level. Then, the following groups: the reducing by lifting and thrusting group, the even reinforcing and reducing group, and the acupuncture were with no operation skills.

### 3.4. Single Factor Repeated Measurement ANOVA

When the same subject was measured at different times (*p*), *p* ≥ 3, the repeated measurement ANOVA is appropriate, because of the possible high correlation between different times. We additionally need to check the covariance matrix sphericity besides the regular ANOVA requirements. Mauchly's test is used to examine sphericity. *P* > 0.05 indicates sphericity reliability, or, the DF of *f*-statistic should be adjusted.  Table 1 is the single factor repeated measurement ANOVA based on the time factor.  Table 2 is based on the manipulation-time factor.

This is the result of each group after the statistics of repeated measurement ANOVA. We can see that after Mauchly's test, the groups dissatisfied with the covariance matrix spherical properties. As we see except the control group, the adjusted *P* < 0.01 in all other groups. The result indicates that the blood perfusion in the other four groups changes with the time changed, and the factor of time plays a different role in different groups.

We can see in this chart that each group is compared with the others, and the operation skills show the difference (*P* < 0.01) expect between even group and no manipulation group. The result indicated that the blood perfusion changes with different acupuncture operation skills.

 In Table 3 are the results of blood perfusion ratio of different manipulation groups at different periods.

From Table 3, we can see that the values of PU in no manipulation group are higher than the control group expect 1 min after acupuncture. The values of PU in 3 different acupuncture manipulation groups increase at all time periods after acupuncture, which have significant statistics difference comparing with the control group. Among the 4 different acupuncture manipulation groups, the values of reinforcing lifting and thrusting group increase at all time periods after acupuncture comparing with the no manipulation group and reducing lifting and thrusting group, and there is a significant statistics difference between the reinforcing lifting and thrusting group and the even reinforcing-reducing group at all time periods after acupuncture. However, the values of reducing lifting and thrusting group are higher than the even reinforcing-reducing group and no manipulation group at all the moments after acupuncture. 

## 4. Discussion

These results indicated that the blood perfusion of Zusanli point was increased after acupuncture. There were different influences on the blood perfusion according to different acupuncture manipulations while the reinforcing manipulation by lifting and thrusting is the most obvious. The reinforcing manipulation can make the blood perfusion kept at a higher level and suggest that this increase may be caused by local vasodilators.

The acupuncture manipulation is one of the most distinctive skills of the acupuncture and moxibustion. The mechanism underlying the acupuncture manipulation is currently unknown. Langevin et al. reported that needle grasp is a measurable biomechanical phenomenon associated with acupuncture manipulation [25] and demonstrated that subtle differences in acupuncture manipulations can affect cellular responses in mouse subcutaneous connective tissue [26]. Furthermore, a study [27] found manual acupuncture could change muscle blood flow locally at different doses and indicated this increase may be caused by local vasodilators. In our country, now most researches focus on the influences of blood biochemical criterions and the function of effect organ by different acupuncture manipulations [10–13].

Actually, acupuncture takes regulating effort by needling the acupoint to stimulate the body regulating system and the key factor is affecting the microenvironment. In our opinion, based on standardization and inheritance of acupuncture manipulation, we observe the mechanism of various manipulations. From our research results, we think acupuncture manipulations may change local acupoint peripheral tissue biophysical characteristics, which is the reason of different influences on the blood perfusion according to different acupuncture manipulations [28].

Whether such blood perfusion changes in acupoints can themselves result in the effectiveness of the acupuncture's therapeutic reinforcing or reducing is at the present unknown. Someone thought the mechanostructural properties of soft connective tissues may affect their response to acupuncture therapy [29]. Dong et al. study indicated that effective acupuncture stimulation is induced mainly due to the receptor deformation [30]. In the future, we will continue our research on the different manipulations from the aspect of blood perfusion, so as to provide more evidence for the mechanism of the acupuncture manipulation.

## Figures and Tables

**Table 1:** Single factor repeated measurement ANOVA (time factor).

	*P*	Mauchly's test	Adjust *P*	
		*P*	G-G	H-F
Reinforcing	<0.01	<0.01	<0.01	<0.01
Reducing	<0.01	<0.01	<0.01	<0.01
Even	<0.01	<0.01	<0.01	<0.01
No manipulation	<0.01	<0.01	<0.01	<0.01
Control	0.31	<0.01	0.390	0.404

**Table 2:** Two-factor repeated measurement ANOVA (manipulation-time factor).

	*F* value	Pr > *F*
Reinforcing-reducing	19.72	<0.01
Reinforcing-even	49.41	<0.01
Reinforcing-acupuncture	73.88	<0.01
Reinforcing-control	135.6	<0.01
Reducing-even	13.4	<0.01
Reducing-acupuncture	30.48	<0.01
Reducing-control	85.15	<0.01
Even-acupuncture	4.803	0.03
Even-control	47.74	<0.01
Acupuncture-control	37.16	<0.01

**Table 3:** Blood perfusion ratio of different manipulation groups at different periods.

Manipulation	Reinforcing	Reducing	Even	Acupuncture	Control
Time	(*N* = 15)	(*N* = 15)	(*N* = 15)	(*N* = 15)	(*N* = 15)
B N	1.0 ± 0.0	1.0 ± 0.0	1.0 ± 0.0	1.0 ± 0.0	1.0 ± 0.0
1 min	2.08 ± 0.29^*⋆*†^	1.71 ± 0.29^*⋆*●^	1.47 ± 0.22^*⋆*●†^	0.99 ± 0.20^●†^	1.01 ± 0.07^●†^
5 min	2.14 ± 0.38^*⋆*†^	1.82 ± 0.36^*⋆*●^	1.47 ± 0.2^*⋆*●†^	1.34 ± 0.13^*⋆*●†^	0.98 ± 0.07^●†^
10 min	2.13 ± 0.37^*⋆*†^	1.73 ± 0.34^*⋆*●^	1.39 ± 0.24^*⋆*●†^	1.30 ± 0.17^*⋆*●†^	0.99 ± 0.09^●†^
15 min	2.03 ± 0.35^*⋆*†^	1.60 ± 0.29^*⋆*●^	1.34 ± 0.26^*⋆*●†^	1.29 ± 0.17^*⋆*●†^	0.97 ± 0.09^●†^
20 min	1.95 ± 0.35^*⋆*†^	1.51 ± 0.29^*⋆*●^	1.30 ± 0.24^*⋆*●†^	1.24 ± 0.14^*⋆*●†^	1.00 ± 0.1^●†^
25 min	1.85 ± 0.35^*⋆*†^	1.43 ± 0.25^*⋆*●^	1.25 ± 0.3^*⋆*●†^	1.24 ± 0.14^*⋆*●†^	0.98 ± 0.1^●†^
30 min	1.77 ± 0.38^*⋆*†^	1.39 ± 0.24^*⋆*●^	1.21 ± 0.17^*⋆*●†^	1.17 ± 0.16^*⋆*●†^	1.00 ± 0.13^●†^

^⋆^Significant difference from control group (*P* < 0.05); ^●^significant difference from reinforcing group (*P* < 0.05); ^†^significant difference from reducing group (*P* < 0.05).

## References

[1] Fisher P, Van Haselen R, Hardy K, Berkovitz S, McCarney R (2004). Effectiveness gaps: a new concept for evaluating health service and research needs applied to complementary and alternative medicine. *Journal of Alternative and Complementary Medicine*.

[2] Silvers M (2000). Acupuncture wins BMA approval. *British Medical Journal*.

[3] Hopton AK, Curnoe S, Kanaan M (2012). Acupuncture in practice: mapping the providers, the patients and the settings in a national cross-sectional survey. *British Medical Journal*.

[4] White A, Foster NE, Cummings M, Barlas P (2007). Acupuncture treatment for chronic knee pain: a systematic review. *Rheumatology*.

[5] Linde K, Allais G, Brinkhaus B, Manheimer E, Vickers A, White AR (2009). Acupuncture for tension-type headache. *Cochrane Database of Systematic Reviews*.

[6] Sherman KJ, Hogeboom CJ, Cherkin DC (2001). How traditional Chinese medicine acupuncturists would diagnose and treat chronic low back pain: results of a survey of licensed acupuncturists in Washington State. *Complementary Therapies in Medicine*.

[7] Langevin HM, Churchill DL, Cipolla MJ (2001). Mechanical signaling through connective tissue: a mechanism for the therapeutic effect of acupuncture. *The FASEB Journal*.

[8] Michael D (2002). Needle manipulation may hold the key to acupuncture’s effects. *Acupuncture Today*.

[9] Kim GH, Yeom M, Yin CS (2010). Acupuncture manipulation enhances antinociceptive effect on for malin-induced pain in rats. *Neurological Research*.

[10] Guo Y (2008). *Experiment Acupuncture Science*.

[11] Li P, Guan W, Wang F (2002). Influence of needling hegu(L14) with twirling reinforcing and reducing manipulation on skin temperature of epigastrium. *Journal of Tianjin College of Traditional Chinese Medicine*.

[12] Wang CH, Wang YP (2007). Influence of different stimulating intensities of twirling manipulation on skin temperature in healthy persons. *Shanghai Journal of Acupuncture and Moxibustion*.

[13] Gao M, Yang HY, Le K (2008). Effects of manual acupuncture and electroacupuncture on Ca^2+^ content and Ca^2+^-ATPase activity in sarcoplasmic reticulum of skeletal muscle cells in rats during acute swimming exercise. *Acupuncture Research*.

[14] Gao XY, Rong PJ, Li L, He W, Ben H, Zhu B (2012). An innovative high-tech acupuncture product: SXDZ-100 nerve muscle stimulator, its theoretical basis, design, and application. *Evidence-Based Complementary and Alternative Medicine*.

[15] Hu Y-E, Yang HY Study of acupuncture manipulation parameter based on data mining technique.

[16] Kubo K, Yajima H, Takayama M, Kebukuro TI, Mizoguchi H, Takakura N (2011). Changes in blood circulation of the contralateralachilles tendon during and after acupuncture. *International Journal of Sports Medicine*.

[17] Kuo TC, Lin CW, Ho FM (2004). The soreness and numbness effect of acupuncture on skin blood flow. *American Journal of Chinese Medicine*.

[18] Hsiu H, Hsu WC, Chen BH, Hsu CL (2010). Differences in the microcirculatory effects of local skin surface contact pressure stimulation between acupoints and nonacupoints: possible relevance to acupressure. *Physiological Measurement*.

[19] Wang GX, Yue CL, Bao J (2007). The influence of electrical acupuncture with different frequency on the blood flow of rats patellar tendon. *Chinese Journal of Sports Medicine*.

[20] Sandberg ML, Sandberg MK, Dahl J (2007). Blood flow changes in the trapezius muscle and overlying skin following transcutaneous electrical nerve stimulation. *Physical Therapy*.

[21] Wang GJ, Han JG, Litscher G, Zhang WB (2012). System identification algorithm analysis of acupuncture effect on mean blood flux of contralateral hegu acupoint. *Evidence-Based Complementary and Alternative Medicine*.

[22] Huang T, Wang RH, Zhang WB (2012). The influence of different methods of acupuncture on skin surface perfusion. *Journal of Traditional Chinese Medicine*.

[23] Hui KKS, Nixon EE, Vangel MG (2007). Characterization of the “deqi” response in acupuncture. *BMC Complementary and Alternative Medicine*.

[24] Leung AY, Park J, Schulteis G, Duann JR, Yaksh T (2006). The electrophysiology of De Qi sensations. *Journal of Alternative and Complementary Medicine*.

[25] Langevin HM, Churchill DL, Fox JR, Badger GJ, Garra BS, Krag MH (2001). Biomechanical response to acupuncture needling in humans. *Journal of Applied Physiology*.

[26] Langevin HM, Bouffard NA, Badger GJ, Churchill DL, Howe AK (2006). Subcutaneous tissue fibroblast cytoskeletal remodeling induced by acupuncture: evidence for a mechanotransduction-based mechanism. *Journal of Cellular Physiology*.

[27] Wang L, Jing MX, Zhi JM, Lu J, Wang CY, Liu QG (2011). Effects of reinforcing and reducing methods by twirling and rotating the needle on contents of CGRP and NO in rats with stressinduced hypertension. *Chinese Acupuncture and Moxibustion*.

[28] Langevin HM, Bouffard NA, Churchill DL, Badger GJ (2007). Connective tissue fibroblast response to acupuncture: dose-dependent effect of bidirectional needle rotation. *Journal of Alternative and Complementary Medicine*.

[29] Kubo K, Yajima H, Takayama M, Ikebukuro T, Mizoguchi H, Takakura N (2008). An in vitro assay of collagen fiber alignment by acupuncture needle rotation. *BioMedical Engineering OnLine*.

[30] Dong Q, Dong X, Li H, Chen D, Xian M (1993). The relations between acupuncture manipulations and responsive discharges of deep receptors. *Acupuncture Research*.

